# Supplementary material for: Sequence variants in ARHGAP15, COLQ and FAM155A associate with diverticular disease and diverticulitis
Source: Nat Commun. 2017 Jun 6;8:15789. doi: 10.1038/ncomms15789 (PMC5467205; doi:10.1038/ncomms15789)
Supplement: Supplementary Information — Supplementary Figures, Supplementary Tables, Supplementary Note, Supplementary Methods and Supplementary References. [file ncomms15789-s1.pdf]

Supplementary Figure 1. Effect of sequence variants on the expression of *ARHGAP15* in white blood cells by RNA sequencing.

a) ***ARHGAP15* transcripts**

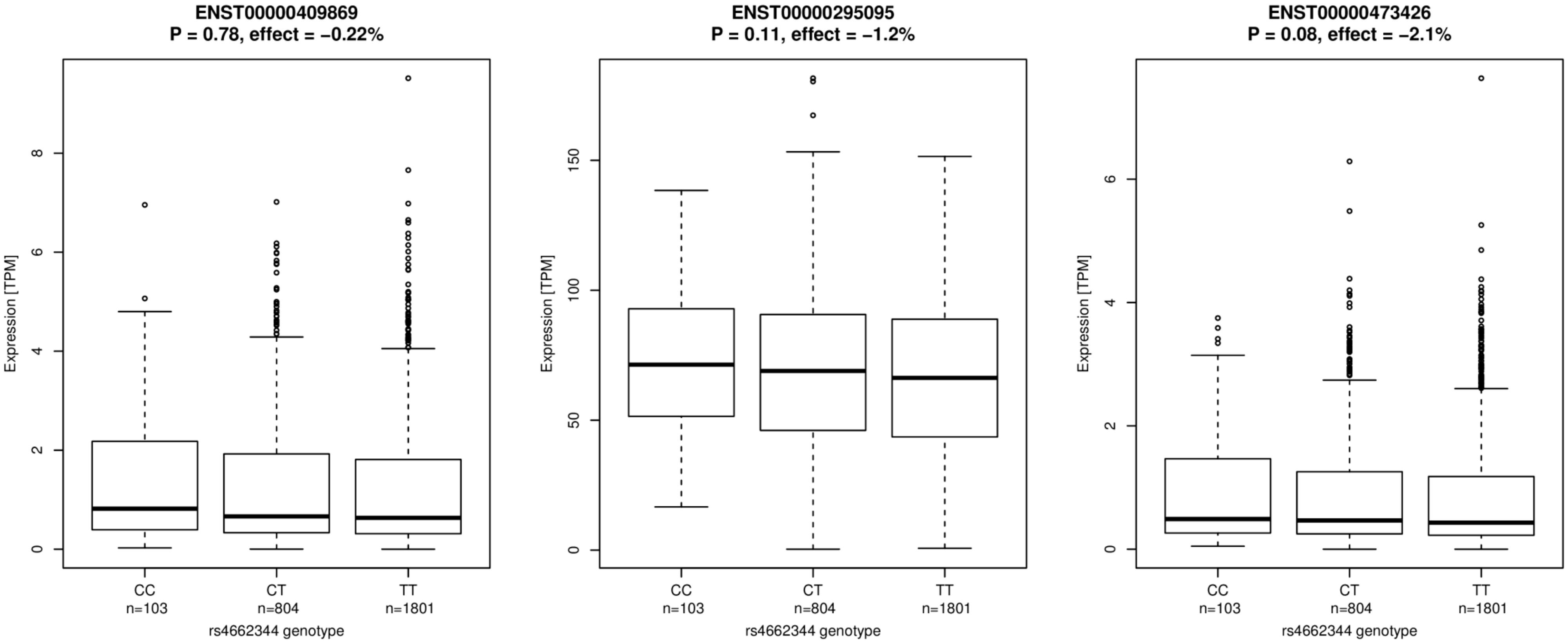

b) *ARHGAP15* exons

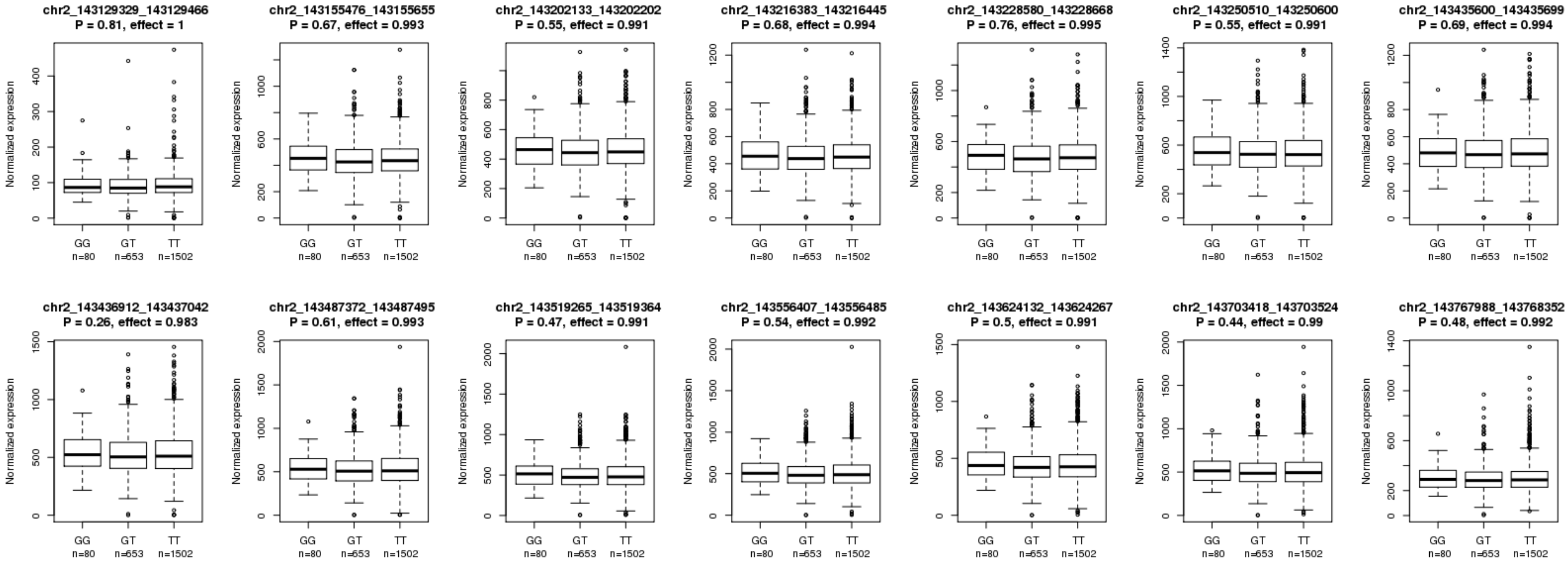

The RNAseq expression in whole blood for different transcripts and each exon is plotted for carriers of wild type, heterozygotes and homozygotes for the associated sequence variant. A line indicates the median, the box 25th and 75th percentiles of the distribution and the whiskers indicate the 95% confidence interval. A) Expression of three *ARHGAP15* transcripts from 2,708 individuals plotted for the sequence variant rs4662344. B) *ARHGAP15* expression, from 2,246 individuals, plotted for the sequence variant rs7607879 (LD with lead SNP r2>0.98)

Supplementary Figure 2. The effect of *ARHGAP15* sequence variants on ROS production.

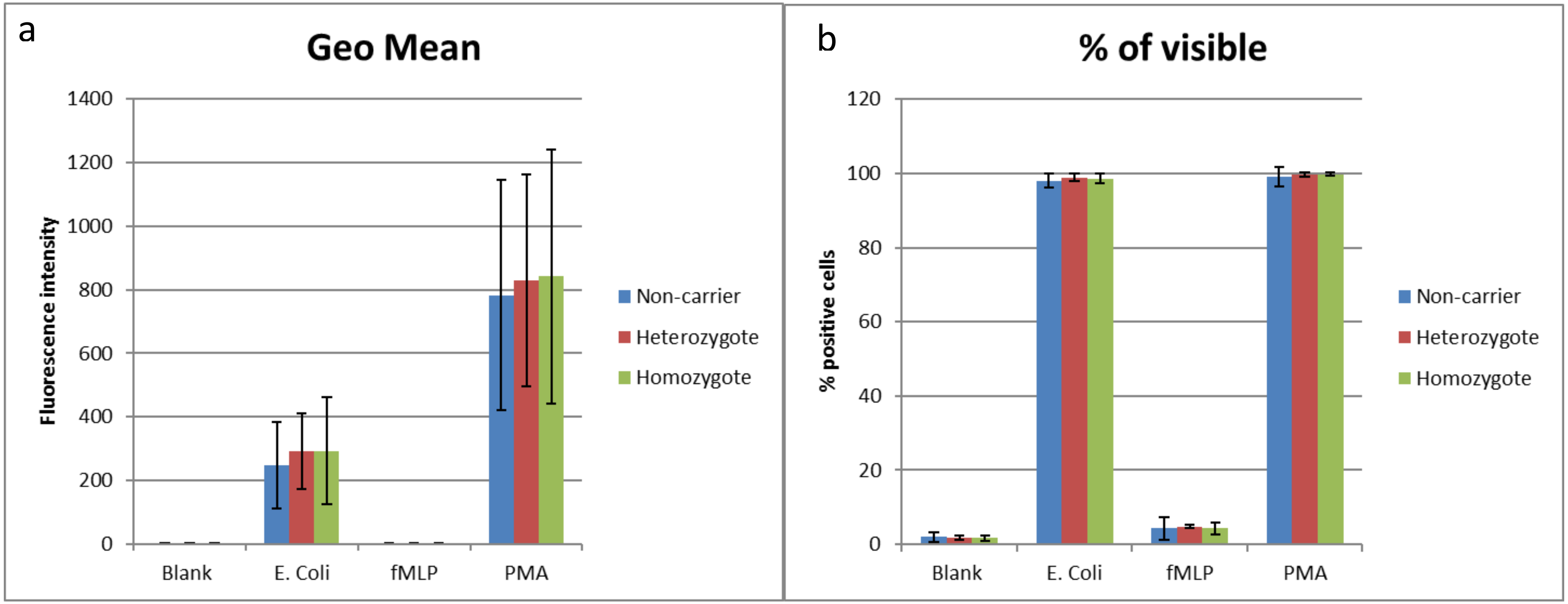

Reactive oxygen species (ROS) production test. Neutrophil respiratory burst after stimulation with: E. Coli, fMLP (Low control) and PMA (High control). Number of blood donors: n=14 non-carriers of the *ARHGAP15* sequence variant rs4662344, n=12 heterozygotes and n=14 homozygotes. A) The average geometric mean of fluorecent intesity for each group of rs4662344 carriers with standard deviation. B) Percentage of ROS positive cells, for each group of rs4662344 carriers, average with standard deviation.

Supplementary Figure 3. Effect of sequence variants on the expression of *COLQ* in white blood cells by RNA sequencing.

a) ***COLQ* transcripts**

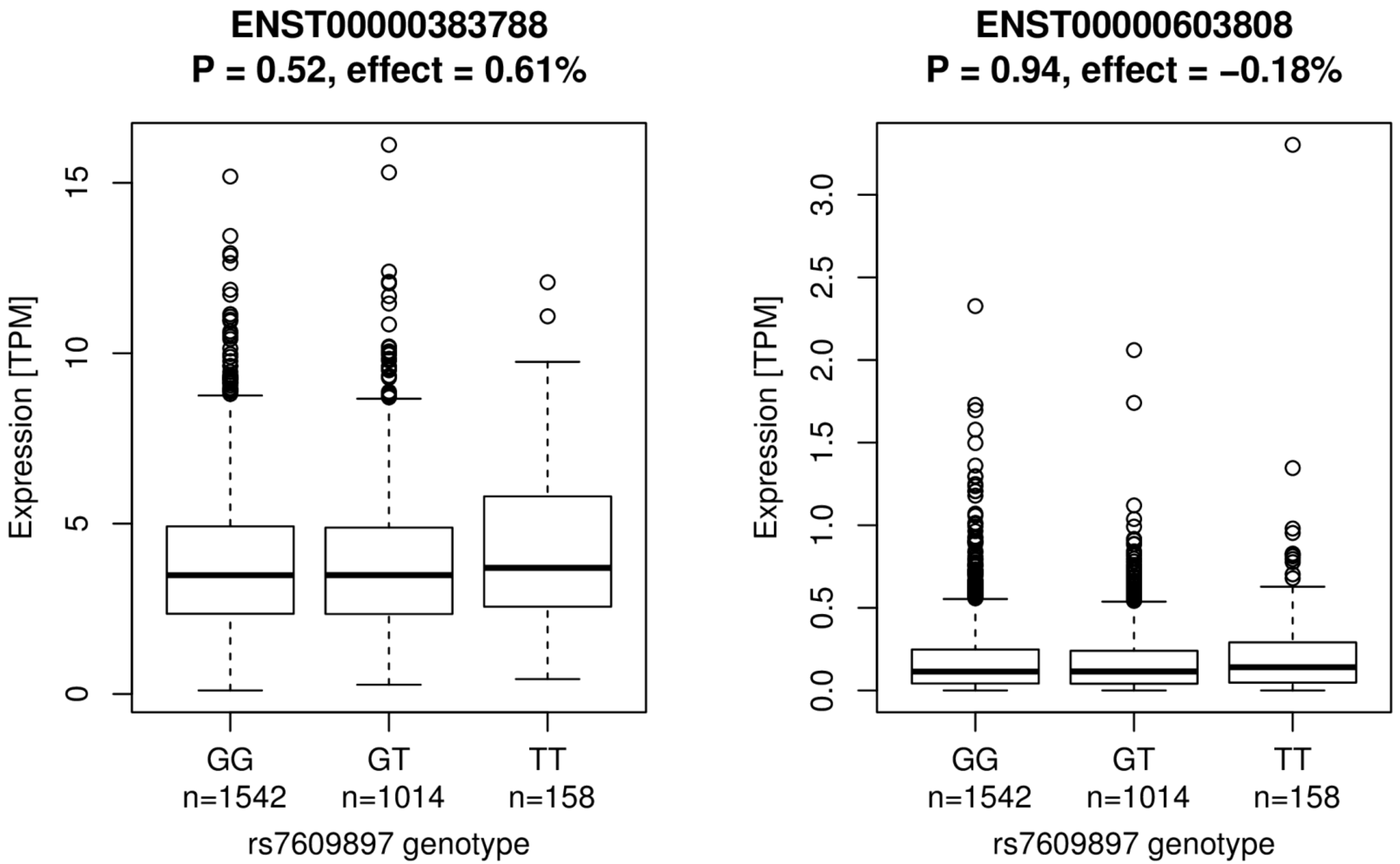

b) *COLQ* exons

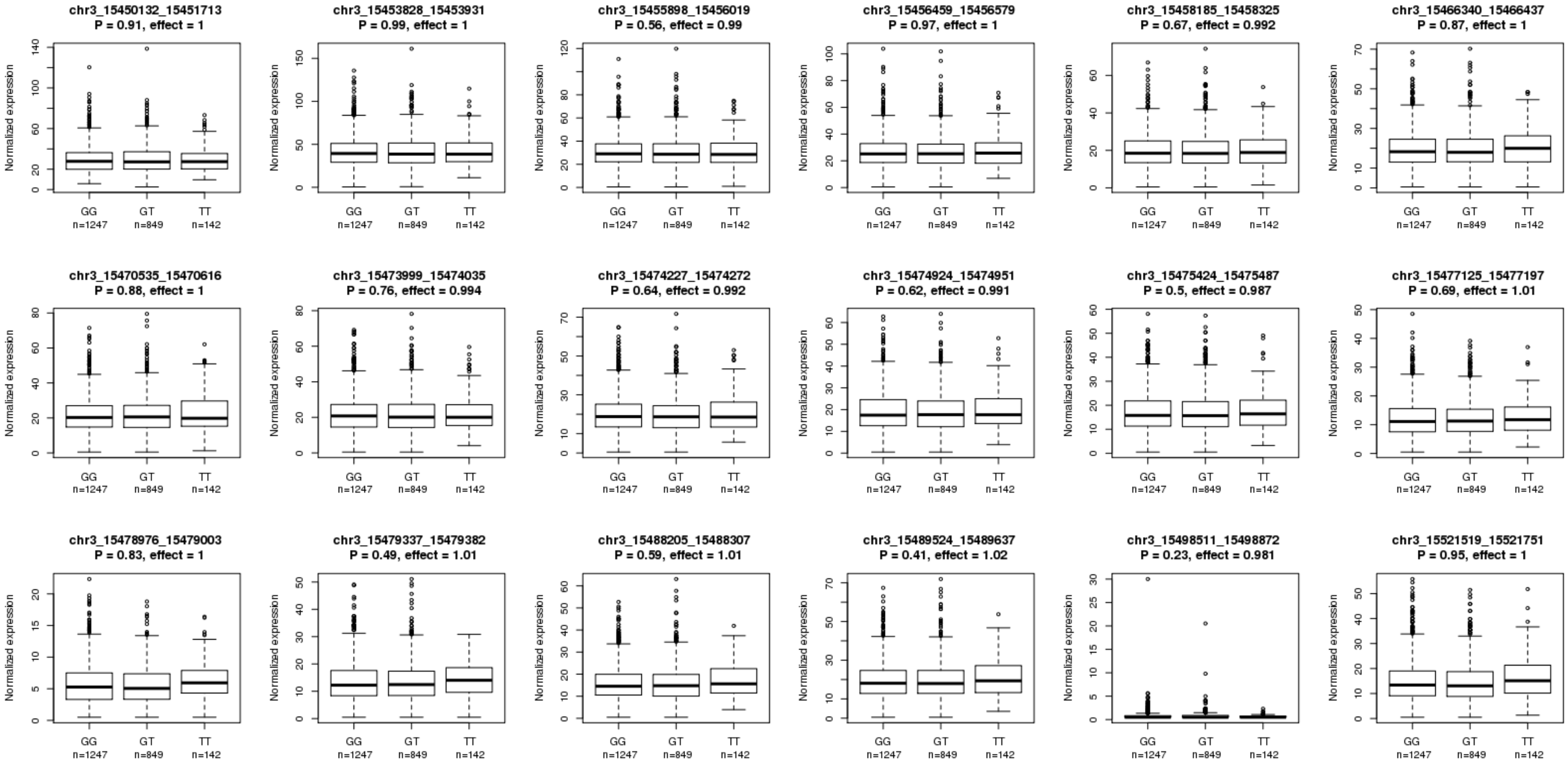

The RNAseq expression in whole blood for each transcript and exon is plotted for carriers of wild type, heterozygotes and homozygotes for the associated sequence variant. A line indicates the median, the box 25th and 75th percentiles of the distribution and the whiskers indicate the 95% confidence interval. A) Expression of two *COLQ* transcripts from 2,708 individuals plotted for the sequence variant rs7609897. B) *COLQ* expression, from 2,246 individuals, plotted for the sequence variant rs7609897.

Supplementary Figure 4. Effect of sequence variants on the expression of *FAM155A* in adipose cells by RNA sequencing.

a) ***FAM155A* transcript**

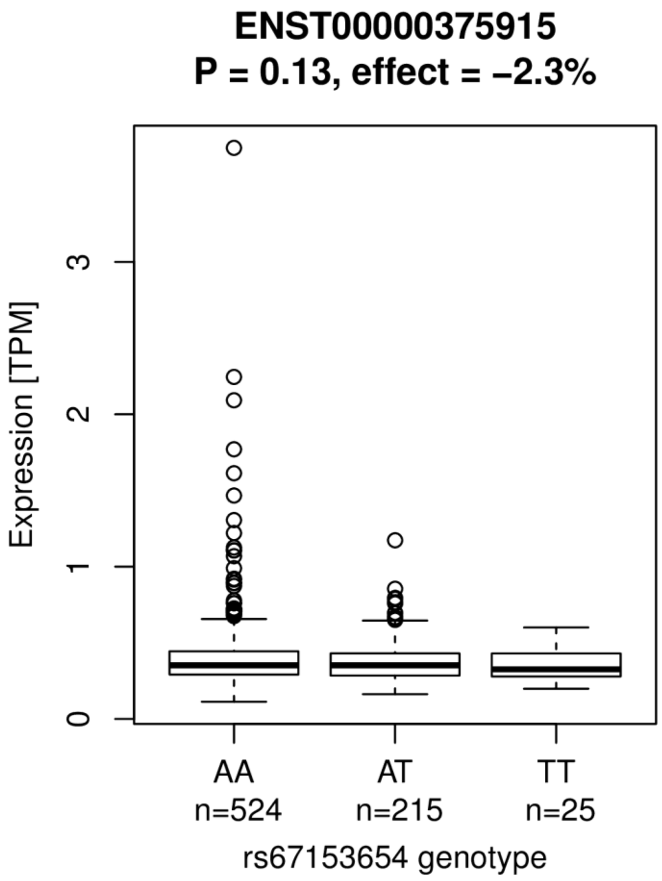

b) ***FAM155A*** **exons**

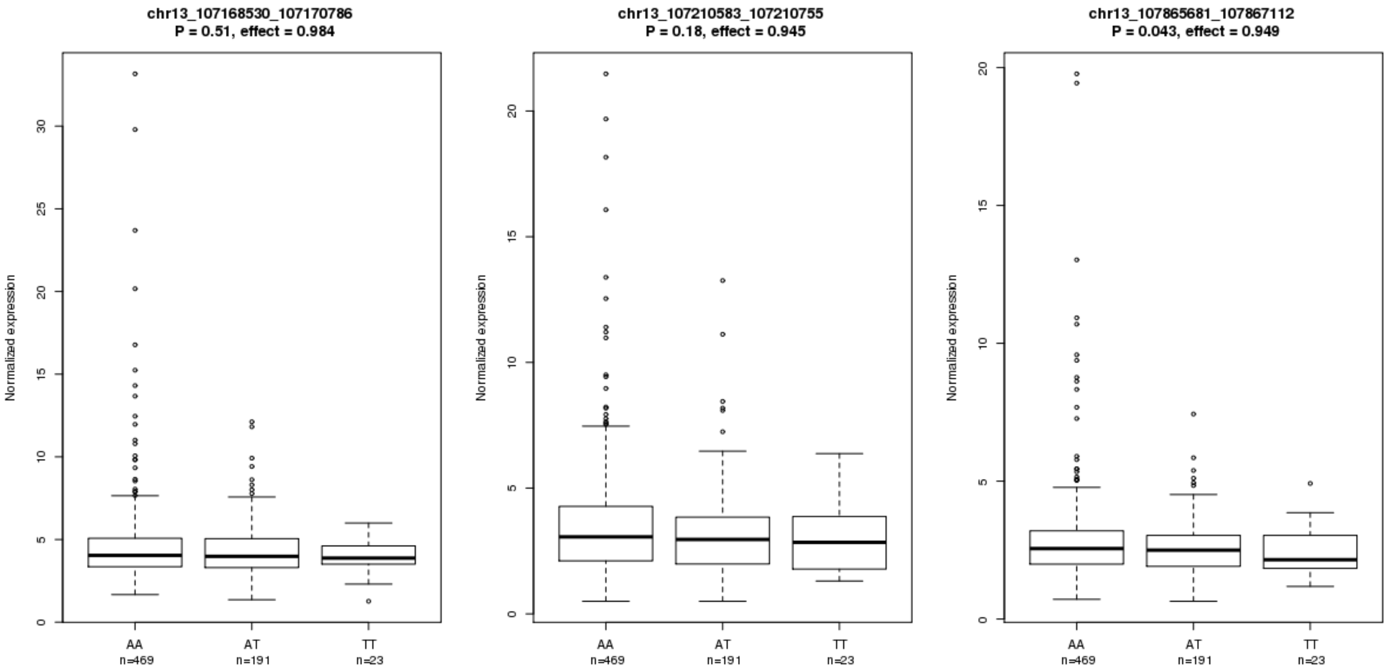

The RNAseq expression in adipose tissue (the gene has very low expression in blood) for each transcript and exon is plotted for carriers of wild type, heterozygotes and homozygotes for the associated sequence variant. A line indicates the median, the box 25th and 75th percentiles of the distribution and the whiskers indicate the 95% confidence interval. A) Expression of one *FAM155A* transcript from 764 individuals. B) *FAM155A* exons are plotted for the sequence variant rs67153654 from 708 individuals.

Supplementary Table 1. The Icelandic diverticular disease cohort.

Covariate	Diverticular disease		Diverticulitis		Uncomplicated diverticular disease		Controls	
	N (% samples with information)	Mean(SD)	N (% samples with information)	Mean(SD)	N (% samples with information)	Mean(SD)	N (% samples with information)	Mean(SD)
**Sex, %females**	5,426 (100%)	59.7%	2,764 (100%)	57.6%	2,662 (100%)	61.9%	245,951	49.3%
**BMI ***	2,891 (55%)	27.8 (5.2)	1,486 (54%)	28.2 (5.1)	1,464 (55%)	27.2 (5.2)	75,939 (31%)	26.3 (5.5)
**Age at diagnosis**	4,841 (91%)	67.7 (14.7)	2,764 (100%)	62.9 (14.8)	2,363 (90%)	73.4 (12.1)	245,951 (100%)	55.4 (19.2)
**%Ever smokers**	2,648 (50%)	82.0%	1,427 (52%)	81.3%	1,266 (48%)	83.0%	70,293 (28.5%)	71.7%

N: Number and percentage of total samples with available information; Age at diagnosis, Body mass index (BMI) and smoking.
*BMI max value was used if multiple measurements were available.

Supplementary Table 2. Bonferroni *P*-value limits for each class of sequence variant.

Variant impact	Definition	N	Bonferroni limit
High	Stop-gain/loss, frameshift, splice donor/acceptor	8,474	2.6×10^{-7}
Medium	Missense, inframe indel, splice region	149,983	5.1×10^{-8}
Low	5'/3'UTR, upstream, downstream, synonymous	2,283,889	4.6×10^{-9}
Other variants - within DHS	Intron, intergenic	3,913,058	2.3×10^{-9}
Other variants - outside DHS	Intron, intergenic	26,108,039	7.9×10^{-10}

N = number of sequence variants in each class. DHS = DNase hypersensitivity sites

Supplementary Table 3a. Sequence variants validated in the Danish diverticular disease sample set.

Diverticular disease

						Icelandic diverticular disease			Danish diverticular disease			Combined Icelandic and Danish diverticular disease sample sets		
						5,426 cases 245,951 controls			5,970 cases, 3,020 controls			11,396 cases 248,971 controls		
SV ID	Position	*P*-value threshold	Nearest gene	SV function	Amin/ Amaj	MAF %	OR (95% ci)	*P*-value	MAF %	OR (95% ci)	*P*-value	OR (95% ci)	*P*-value	P het
rs4662344	chr2:143591289	2.3×10^{-9}	*ARHGAP15*	intron	T/C	20.9/17.7	1.23 (1.16,1.31)	$4.9x10^{-13}$	21.7/18.5	1.22 (1.13,1.32)	$7.0x10^{-7}$	1.23 (1.17,1.29)	$1.9x10^{-18}$	0.86
rs7609897	chr3:15461174	2.3×10^{-9}	*COLQ*	intron	T/G	22.1/24.7	0.85 (0.80,0.89)	$1.6x10^{-9}$	21.6/23.3	0.91 (0.84,0.98)	$1.0x10^{-2}$	0.87 (0.83,0.91)	$1.5x10^{-10}$	0.17
rs67153654	chr13:107572636	2.3×10^{-9}	*FAM155A*	intron	A/T	17.0/18.6	0.89 (0.84,0.94)	$8.7x10^{-5}$	17.4/20.1	0.84 (0.78,0.91)	$2.2x10^{-5}$	0.87 (0.83,0.91)	$1.3x10^{-8}$	0.30
rs61756577	chr7:43492135	5.1×10^{-8}	*HECW1*	missense	C/G	0.98/0.64	1.74 (1.37,2.19)	$3.9x10^{-6}$	0.25/0.13	1.84 (0.87,3.86)	$1.1x10^{-1}$	1.74 (1.40,2.18)	$1.0x10^{-6}$	0.89
rs9514637	chr13:107249894	7.9×10^{-10}	*FAM155A*	intron	A/G	24.9/27.6	0.89 (0.85,0.94)	$1.6x10^{-5}$	23.6/24.2	0.97 (0.90,1.04)	0.38	0.92 (0.88,0.96)	$5.6x10^{-5}$	0.077
rs2272901	chr6:149218637	4.6×10^{-9}	*TAB2*	5 prime UTR	A/G	25.8/28.0	0.90 (0.85,0.95)	$4.1x10^{-5}$	26.6/27.7	0.95 (0.88,1.02)	0.13	0.92 (0.88,0.95)	$2.4x10^{-5}$	0.24
rs761545809	chr6:136194522	4.6×10^{-9}	*PDE7B*	3 prime UTR	A/AAAG	0.44/0.22	2.13 (1.45,3.12)	$1.1x10^{-4}$	0.08/0.1	0.84 (0.19,3.77)	0.82	2.01 (1.39,2.91)	$2.2x10^{-4}$	0.24
rs1945615	chr11:15081931	4.6×10^{-9}	*CALCB*	downstream	A/G	32.7/30.2	1.1 (1.05,1.15)	$1.5x10^{-4}$	31.0/30.1	1.04 (0.97,1.11)	0.24	1.08 (1.04,1.12)	$1.7x10^{-4}$	0.21
rs17636410	chr9:72401105	1.1×10^{-9}	.	intergenic	A/G	27.3/25.1	1.11 (1.06,1.17)	$4.6x10^{-5}$	25.2/24.8	1.02 (0.95,1.10)	0.60	1.08 (1.04,1.13)	$2.8x10^{-4}$	0.055
rs11263878	chr1:36392266	4.6×10^{-9}	*LSM10*	downstream	T/C	16.6/17.9	0.93 (0.87,0.98)	0.011	17.1/17.4	0.98 (0.90,1.07)	0.62	0.94 (0.90,0.99)	0.018	0.30
rs61741212	chr19:35666838	5.1×10^{-8}	*UPK1A*	missense	T/C	8.57/9.99	0.81 (0.75,0.88)	$3.7x10^{-7}$	12.3/11.3	1.11 (1.00,1.22)	0.041	0.92 (0.87,0.98)	$7.8x10^{-3}$	$1.7x10^{-6}$
rs140894490	chr7:44189408	4.6×10^{-9}	*GCK*	5 prime UTR	C/T	0.68/0.39	2.17 (1.63,2.88)	$1.0x10^{-7}$	0.18/0.25	1.04 (0.96,1.13)	0.33	1.1 (1.02,1.18)	0.018	$1.1x10^{-6}$
rs149367812	chr1:186367896	5.1×10^{-8}	*TPR*	missense	A/T	0.42/0.2	1.84 (1.23,2.76)	$3.1x10^{-3}$	0.48/0.48	1 (0.63,1.58)	0.99	1.41 (1.04,1.91)	0.027	0.049
rs143318100	chr1:154348533	5.1×10^{-8}	*ATP8B2*	missense	A/G	0.19/0.27	0.74 (0.46,1.19)	0.21	0.01/0.02	0.51 (0.03,8.29)	0.63	0.73 (0.46,1.17)	0.19	0.79
rs773564372	chr7:43558103	7.9×10^{-10}	*HECW1*	intron	T/C	0.71/0.38	2.32 (1.75, 3.07)	4.9×10^{-9}	np	np	np	np	np	np
rs760491413	chr1:235999377	7.9×10^{-10}	*NID1*	intron	A/C	0.23/0.05	5.48 (2.74, 10.97)	1.6×10^{-6}	np	np	np	np	np	np

SV ID, Sequence variant identification. Position on the hg38. Amin, minor allele; Amaj, major allele; MAF, Minor allele frequency. MAF is calculated on the chip typed samples, excluding familialy imputed genotypes. OR, odds ratio of the minor allele; 95% CI, 95% confidence interval. np = not polymorphic, threshold for genome wide significance for an intronic-variant is $P< 2.3x10^{-9}$. P het, *P*-value for the heterogeneity between cohorts.

Supplementary Table 3b. Sequence variants validated in the Danish diverticular disease sample set.

Diverticulitis

						Icelandic Diverticulitis 2,764 cases 245,951 controls			Danish diverticular disease 5,970 cases, 3,020 controls			Combined Icelandic diverticulitis and Danish diverticular disease sample sets 8,734 cases 248,971 controls		
**SV ID**	**Position**	***P*-value threshold**	**Nearest gene**	**SV function**	**Amin/Amaj**	**MAF %**	**OR (95% ci)**	***P*-value**	**MAF %**	**OR (95% ci)**	***P*-value**	**OR (95% ci)**	***P*-value**	**P het**
rs4662344	chr2:143591289	2.3×10^{-9}	*ARHGAP15*	intron	T/C	21.3/17.7	1.26 (1.16,1.36)	$4.5\text{x}10^{-9}$	21.7/18.5	1.22 (1.13,1.32)	$7.0\text{x}10^{-7}$	1.24 (1.17,1.31)	$1.8\text{x}10^{-14}$	0.62
rs7609897	chr3:15461174	2.3×10^{-9}	*COLQ*	intron	T/G	21.0/24.7	0.80 (0.74,0.86)	$1.9\text{x}10^{-9}$	21.6/23.3	0.91 (0.84,0.98)	$1.0\text{x}10^{-2}$	0.85 (0.80,0.89)	$1.0\text{x}10^{-9}$	0.02
rs67153654	chr13:107572636	2.3×10^{-9}	*FAM155A*	intron	A/T	15.6/18.6	0.80 (0.74,0.87)	$2.3\text{x}10^{-7}$	17.4/20.1	0.84 (0.78,0.91)	$2.2\text{x}10^{-5}$	0.82 (0.78,0.87)	$3.0\text{x}10^{-11}$	0.43
rs61756577	chr7:43492135	5.1×10^{-8}	*HECW1*	missense	C/G	0.91/0.64	1.73 (1.25,2.37)	$8.3\text{x}10^{-4}$	0.25/0.13	1.84 (0.87,3.86)	0.11	1.74 (1.30,2.34)	$2.1\text{x}10^{-4}$	0.88
rs9514637	chr13:107249894	7.9×10^{-10}	*FAM155A*	intron	A/G	24.4/27.6	0.82 (0.76,0.88)	$5.0\text{x}10^{-8}$	23.6/24.2	0.97 (0.90,1.04)	0.38	0.89 (0.85,0.94)	$6.2\text{x}10^{-6}$	$1.5\text{x}10^{-3}$
rs2272901	chr6:149218637	4.6×10^{-9}	*TAB2*	5 prime UTR	A/G	24.5/27.9	0.84 (0.78,0.90)	$5.8\text{x}10^{-7}$	26.6/27.7	0.95 (0.88,1.02)	0.13	0.89 (0.85,0.94)	$3.8\text{x}10^{-6}$	0.015
rs761545809	chr6:136194522	4.6×10^{-9}	*PDE7B*	3 prime UTR	A/AAAG	0.58/0.22	3.06 (1.94,4.81)	$1.3\text{x}10^{-6}$	0.08/0.1	0.84 (0.19,3.77)	0.82	2.74 (1.78,4.24)	$5.1\text{x}10^{-6}$	0.11
rs1945615	chr11:15081931	4.6×10^{-9}	*CALCB*	downstream	A/G	34.3/30.2	1.18 (1.11,1.26)	$4.3\text{x}10^{-7}$	31.0/30.1	1.04 (0.97,1.11)	0.24	1.11 (1.06,1.17)	$8.8\text{x}10^{-6}$	$7.5\text{x}10^{-3}$
rs17636410	chr9:72401105	1.1×10^{-9}	.	intergenic	A/G	28.9/25.1	1.19 (1.11,1.28)	$6.5\text{x}10^{-7}$	25.2/24.8	1.02 (0.95,1.10)	0.60	1.11 (1.05,1.16)	$7.3\text{x}10^{-5}$	$2.3\text{x}10^{-3}$
rs11263878	chr1:36392266	4.6×10^{-9}	*LSM10*	downstream	T/C	14.7/17.9	0.82 (0.75,0.89)	$2.1\text{x}10^{-6}$	17.1/17.4	0.98 (0.90,1.07)	0.62	0.89 (0.84,0.95)	$1.7\text{x}10^{-4}$	$3.3\text{x}10^{-3}$
rs61741212	chr19:35666838	5.1×10^{-8}	*UPK1A*	missense	T/C	8.05/9.99	0.79 (0.71,0.88)	$1.6\text{x}10^{-5}$	12.3/11.3	1.11 (1.00,1.22)	0.04	0.95 (0.88,1.02)	0.17	$4.8\text{x}10^{-6}$
rs140894490	chr7:44189408	4.6×10^{-9}	*GCK*	5 prime UTR	C/T	0.59/0.39	2.01 (1.35,2.98)	$5.2\text{x}10^{-4}$	0.18/0.25	1.04 (0.96,1.13)	0.33	1.07 (0.99,1.15)	0.10	$1.3\text{x}10^{-3}$
rs149367812	chr1:186367896	5.1×10^{-8}	*TPR*	missense	A/T	0.54/0.2	2.44 (1.48,4.01)	$4.3\text{x}10^{-4}$	0.48/0.48	1 (0.63,1.58)	0.99	1.51 (1.07,2.11)	0.017	$9.5\text{x}10^{-3}$
rs143318100	chr1:154348533	5.1×10^{-8}	*ATP8B2*	missense	A/G	0.0/0.27	0.16 (0.05,0.44)	$4.9\text{x}10^{-4}$	0.01/0.02	0.51 (0.03,8.29)	0.63	0.18 (0.07,0.48)	$5.9\text{x}10^{-4}$	0.44
rs773564372	chr7:43558103	7.9×10^{-10}	*HECW1*	intron	T/C	0.60/0.38	2.06 (1.39,3.06)	3.5×10^{-4}	np	np	np	np	np	Np
rs760491413	chr1:235999377	7.9×10^{-10}	*NID1*	intron	A/C	0.29/0.05	6.99 (3.11,15.7)	2.6×10^{-6}	np	np	np	np	np	Np

SV ID, Sequence variant identification. Position on the hg38. Amin, minor allele; Amaj, major allele; MAF, Minor allele frequency. MAF is calculated on the chip typed samples only, excluding familialy imputed genotypes. OR, odds ratio of the minor allele; 95% CI, 95% confidence interval. np = not polymorphic, threshold for genome wide significance for an intronic-variant is $P< 2.3\text{x}10^{-9}$. P het, *P*-value for the heterogeneity between cohorts.

Supplementary Table 3c. Sequence variants validated in the Danish diverticular disease sample set.

Uncomplicated diverticular disease

						Icelandic uncomplicated diverticular disease<br>2,662 cases, 245,951 controls			Danish diverticular disease<br>5,970 cases, 3,020 controls			Combined Icelandic uncomplicated diverticular disease and Danish diverticular disease sample sets<br>8,632 cases 248,971 controls		
**SV ID**	**Position**	***P*-value threshold**	**Nearest gene**	**SV function**	**Amin/Amaj**	**MAF %**	**OR (95% ci)**	***P*-value**	**MAF %**	**OR (95% ci)**	***P*-value**	**OR (95% ci)**	***P*-value**	**P het**
rs4662344	chr2:143591289	2.3×10^{-9}	*ARHGAP15*	intron	T/C	20.4/17.7	1.2 (1.11,1.30)	$2.6x10^{-6}$	21.7/18.5	1.22 (1.13,1.32)	$7.0x10^{-7}$	1.21 (1.15,1.28)	$8.6x10^{-12}$	0.77
rs7609897	chr3:15461174	2.3×10^{-9}	*COLQ*	intron	T/G	23.7/24.7	0.91 (0.84,0.97)	$6.1x10^{-3}$	21.6/23.3	0.91 (0.84,0.98)	0.01	0.91 (0.86,0.95)	$1.7x10^{-4}$	1
rs67153654	chr13:107572636	2.3×10^{-9}	*FAM155A*	intron	A/T	18.87/18.6	0.99 (0.91,1.06)	0.72	17.4/20.1	0.84 (0.78,0.91)	$2.2x10^{-5}$	0.91 (0.87,0.97)	$1.4x10^{-3}$	$4.9x10^{-3}$
rs61756577	chr7:43492135	5.1×10^{-8}	*HECW1*	missense	C/G	1.01/0.64	1.74 (1.27,2.38)	$5.2x10^{-4}$	0.25/0.13	1.84 (0.87,3.86)	0.11	1.75 (1.31,2.34)	$1.3x10^{-4}$	0.89
rs9514637	chr13:107249894	7.9×10^{-10}	*FAM155A*	intron	A/G	25.8/27.6	0.98 (0.91,1.05)	0.49	23.6/24.2	0.97 (0.90,1.04)	0.38	0.97 (0.92,1.02)	0.27	0.87
rs2272901	chr6:149218637	4.6×10^{-9}	*TAB2*	5 prime UTR	A/G	27.6/27.95	0.97 (0.91,1.04)	0.40	26.6/27.7	0.95 (0.88,1.02)	0.13	0.96 (0.91,1.01)	0.094	0.61
rs761545809	chr6:136194522	4.6×10^{-9}	*PDE7B*	3 prime UTR	A/AAAG	0.27/0.22	1.15 (0.61,2.15)	0.67	0.08/0.1	0.84 (0.19,3.77)	0.82	1.1 (0.61,1.96)	0.76	0.71
rs1945615	chr11:15081931	4.6×10^{-9}	*CALCB*	downstream	A/G	30.8/30.2	1.01 (0.94,1.08)	0.79	31.0/30.1	1.04 (0.97,1.11)	0.24	1.02 (0.98,1.07)	0.31	0.52
rs17636410	chr9:72401105	1.1×10^{-9}	.	intergenic	A/G	25.3/25.1	1.03 (0.96,1.10)	0.43	25.2/24.8	1.02 (0.95,1.10)	0.60	1.02 (0.97,1.08)	0.35	0.87
rs11263878	chr1:36392266	4.6×10^{-9}	*LSM10*	downstream	T/C	19/17.9	1.05 (0.97,1.14)	0.21	17.1/17.4	0.98 (0.90,1.07)	0.62	1.02 (0.96,1.08)	0.55	0.22
rs61741212	chr19:35666838	5.1×10^{-8}	*UPK1A*	missense	T/C	9.25/9.99	0.85 (0.76,0.94)	$2.1x10^{-3}$	12.3/11.3	1.11 (1.00,1.22)	0.041	0.98 (0.91,1.05)	0.55	$2.6x10^{-4}$
rs140894490	chr7:44189408	4.6×10^{-9}	*GCK*	5 prime UTR	C/T	0.75/0.39	2.34 (1.61,3.41)	$9.5x10^{-6}$	0.18/0.25	1.04 (0.96,1.13)	$3.3x10^{-1}$	1.08 (1.00,1.16)	0.062	$3.6x10^{-5}$
rs149367812	chr1:186367896	5.1×10^{-8}	*TPR*	missense	A/T	0.27/0.2	1.17 (0.63,2.19)	0.62	0.48/0.48	1 (0.63,1.58)	0.99	1.05 (0.73,1.53)	0.78	0.68
rs143318100	chr1:154348533	5.1×10^{-8}	*ATP8B2*	missense	A/G	0.41/0.27	1.36 (0.81,2.31)	0.25	0.01/0.02	0.51 (0.03,8.29)	0.63	1.32 (0.79,2.21)	0.29	0.49
rs773564372	chr7:43558103	7.9×10^{-10}	*HECW1*	intron	T/C	0.81/0.38	2.4 (1.66,3.47)	3.0×10^{-6}	np	np	np	np	np	np
rs760491413	chr1:235999377	7.9×10^{-10}	*NID1*	intron	A/C	0.14/0.05	2.7 (0.87,8.39)	0.086	np	np	np	np	np	np

SV ID, Sequence variant identification. Position on the hg38. Amin, minor allele; Amaj, major allele; MAF, Minor allele frequency. MAF is calculated on the chip typed samples only, excluding familialy imputed genotypes. OR, odds ratio of the minor allele; 95% CI, 95% confidence interval. np = not polymorphic, threshold for genome wide significance for an intronic-variant is $P < 2.3x10^{-9}$. P het, *P*-value for the heterogeneity between cohorts.

Supplementary Table 4. Diverticulitis vs uncomplicated diverticular disease.

**Gene**	**SV ID**	**OR (95% CI)**	***P*-value**
ARHGAP15	rs4662344	1.01 (0.90,1.13)	0.90
COLQ	rs7609897	0.93 (0.83,1.03)	0.17
FAM155A	rs67153654	0.84 (0.74,0.94)	$3.8x10^{-3}$

SV ID, Sequence variant identification. OR, odds ratio of the minor allele; 95% CI, 95% confidence interval. Diverticulitis N=2,748 Diverticular disease, without known diverticulitis N= 2,662

Supplementary Table 5. Genes and missense/LOF variants within each locus (+/- 100kb of lead sequence variant).

ARHGAP15

Genes	# LOF	*P*-value*	# missense	*P*-value*	Gene name	GTEx tissue expression, top 5	UniProtKB short description
ARHGAP15	0	-	9	0.507	Rho GTPase activating protein 15	Whole Blood, EBV transformed lymphocytes, Spleen, Nerve-Tibial,Small Intestine -Terminal Ileum	GTPase activator for the Rho-type GTPases by converting them to an inactive GD*P*-bound state. Has activity toward RAC1. Overexpression results in an increase in actin stress fibers and cell contraction

COLQ

Genes	# LOF	*P*-value*	# missense	*P*-value*	Gene name	GTEx tissue expression, top 5	UniProtKB short description
COLQ	0	-	12	$2.3x10^{-4}$	Collagen-like tail subunit (single strand of homotrimer) of asymmetric acetylcholinesterase	Brain-cerebellar Hemisphere, Brain-cerebellum, Testis, Spleen, Heart-Atrial appendage	Anchors the catalytic subunits of asymmetric AChE to the synaptic basal lamina
HACL1	2	0.6789	11	0.042	2-hydroxyacyl-CoA lyase 1	Testis, EBV transformed lymphocytes, Adipose, Thyroid, Breast mammary tissue	Catalyzes a carbon-carbon cleavage reaction; cleaves a 2-hydroxy-3-methylacyl-CoA into formyl-CoA and a 2-methyl-branched fatty aldehyde
EAF1	0	-	6	0.32	ELL associated factor 1	EBV transformed lymphocytes,Testis, Brain-cerebellar Hemisphere, Pituitary,Brain-Frontal Cortex	Acts as a transcriptional transactivator of ELL and ELL2 elongation activities
METTL6	1	0.07142	4	0.071	Methyltransferase like 6	Testis, Brain-Cerebellar Hemisphere,Brain-Cerebellum, Brain-Frontal Cortex, Cells transformed fibroblasts	Probable methyltransferase

FAM155A

Genes	# LOF	*P*-value*	# missense	*P*-value*	Gene name	GTEx tissue expression, top 5	UniProtKB short description
FAM155A	0	-	16	0.094	Family with sequence similarity 155, member A	Pituitary, Brain-Frontal Cortex, Brain-Hypothalamus, Brain-Anterior cingulate cortex, Brain-Cortex	na

* *P*-value for the association for the strongest variant. For each locus (+/- 100 kb of lead association variant) all protein coding genes are listed with the number of loss of function (LOF) and missense/splice sequence variants in each gene. The best *P*-value for association with diverticular disease from a coding variant in each gene is given. The five tissues with the highest expression according to GTEx are listed, and a short description of each gene according to UniProtKB database.

Supplementary Table 6. IBD Polygenic Risk Score analysis.

	P < $5x10^{-8}$		P < 0.05	
**Polygenic risk score for inflammatory bowel disease**				
	***P*-value**	**R^2 (%)**	***P*-value**	**R^2 (%)**
Crohns disease	$4.11x10^{-22}$	2.5	$2.15x10^{-29}$	3.4
Diverticular disease	0.27	0.002	0.083	0.0063
Diverticulitis	0.35	0.0045	0.3	0.0047
Inflammatory Bowel Disease	$1.26x10^{-86}$	2.9	$7.39x10^{-104}$	3.5
Ulcerative Colitis	$1.17x10^{-63}$	2.6	$9.98x10^{-76}$	3.1
**Polygenic risk score for Crohn‘s Disease**				
	***P*-value**	**R^2 (%)**	***P*-value**	**R^2 (%)**
Crohns disease	$9x10^{-27}$	3.1	$3.51x10^{-30}$	3.5
Diverticular disease	0.16	0.0039	0.34	0.00084
Diverticulitis	0.54	0.0014	0.45	0.0017
Inflammatory Bowel Disease	$1.66x10^{-56}$	1.9	$2.2x10^{-68}$	2.2
Ulcerative Colitis	$1.88x10^{-34}$	1.4	$2.44x10^{-43}$	1.7
**Polygenic risk score for Ulcerative colitis**				
	***P*-value**	**R^2 (%)**	***P*-value**	**R^2 (%)**
Crohns disease	$2.09E^{-08}$	0.87	$1.70E^{-16}$	1.8
Diverticular disease	0.34	0.0014	0.092	0.0097
Diverticulitis	0.53	0.0024	0.24	0.0093
Inflammatory Bowel Disease	$1.98x10^{-83}$	2.8	$8.23x10^{-101}$	3.4
Ulcerative Colitis	$9.96x10^{-76}$	3.1	$1.41x10^{-84}$	3.5

Polygenic risk scores (PRS) were calculated using publicly available summary statistics of IBD/UC/CD in Europeans from an Immunochip GWAS study on IBD [2]. Polygenic risk scores were then tested for association with diverticular disease and diverticulitis using generalized additive regression with smoothed age, sex and the first five principal components as covariates using *P*-value threshold of P <$5x10^{-8}$ and P<0.05. R^2 (%): Variance explained was estimated using Nagelkerke‘s pseudo R-squared.

Supplementary Table 7. Sequence variants previously reported to associate to diverticular disease or related diseases.

Gene	SNP	*P*-value	OR (95%CI)
RPRM	rs1063728	0.98	1.00 (0.92,1.09)
TNFSF15	rs7848647	0.49	1.02 (0.97,1.07
COL3A1	rs1800255	0.74	1.01 (0.96,1.06)

P-value and odds ratio (OR) with 95% CI, 95% confidence interval for the association with diverticular disease in Iceland.

Supplementary Table 8. Haploreg analysis of SNPs in LD (r2>0.6) with the lead associated sequence variants.

Genes	variant	Ref	Alt	SiPhy cons	Promoter histone marks	Enhancer histone marks	DNAse	Motifs changed
ARHGAP15	rs61603193	AT	A	yes	FAT, STRM, VAS, SKIN, BONE	BLOOD, STRM, MUS, SKIN, FAT, GI, LUNG, BRAIN	ESDR,LUNG,GI, MUS,SKIN	Cdx,Foxa,Foxp1,Hoxa9, Sox,Zfp105
COLQ	rs7609897	G	T	no		BLOOD, HEART, THYM, PANC		BATF,Nrf1
FAM155A	rs67153654	T	A	no	BRAIN	ESDR	BRAIN	Nanog,Sox,Zfp105

Results from Haploreg 4.1 August 2016. URL:http://www.broadinstitute.org/mammals/haploreg/haploreg.php

Supplementary note.

We did not find any effect of rs4662344-T (or other variants associating with diverticular disease) at the *ARHGAP15* locus on expression in blood or adipocytes. We looked for potential effects of these variants on transcription factor and enhancer regions. H3K27 acetylation, DNase I sensitivity and the SiPhy conservation score indicate that the indel rs61603193 (AT/A, r^2=0.97 with rs4662344-T) could be in an enhancer region (Haploreg 4.1)[3] (Supplementary Table 5). Acetylation marks are observed in multiple tissues including colon smooth muscle, sigmoid colon and colonic mucosa and rs61603193 alters six potential transcription factor (TF) binding sites, with the largest effect on Cdx, the caudal-related homeodomain TFs. Cdx1 and Cdx2 are expressed in colon and Cdx2 is required for intestinal development and colon specification in mice[4, 5].

rs7609897-T in *COLQ* is in an intron with potential histone marks in four tissues, including primary T-cells and could introduce regulatory motifs for BATF_disc3 and Nrf1_known2 (Haploreg 4.1[3]).

At the *FAM155A* (Family With Sequence Similarity 155A) locus there are fourteen variants in LD (r^2>0.6) with rs67153654-A, which has the potential to disrupt regulatory motifs for Sox_13, Sox_10 and Nanog_disc2 and has histone modification signals in brain and neuronal cells (Haploreg 4.1[3]).

The best coding signal identified in this study is represented by a rare missense variant rs61756577-C (p.Val1065Leu, MAF 0.65%) in HECW1 (OR=1.74, P=3.9×10^{-6}) and a similar effect in the Danish sample (OR=1.84, P=0.11, P_{het}=0.90), but association in Icelandic and Danish samples combined (OR=1.74, P=1.0×10^{-6}) did not reach genome-wide significance (Supplementary Table 3a). The *HECW1* gene also harbors 8 rare intronic variants, marked by rs77356437 (MAF 0.40%) showing suggestive association with diverticular disease (OR=2.32, P=4.9×10^{-9}). rs77356437 correlated with the HECW1 missense variant rs61756577-C (r^2=0.68). However, rs77356437 is not present in the Danish samples and thus the association could not be validated (Supplementary Table 2a).

Supplementary methods.

Effect of smoking.

We used questionnaire data to assess whether smoking affects the risk of DD in Iceland[6, 7]. Heavy smokers defined to have 10 pack years or more (N=26,113, >10 pack-years; 1 packyear defined as 1 pack of cigarettes per day for 1 year)

were compared to those who answered that they had never smoked (N=22,815). The risk of being diagnosed with DD was calculated for each group and adjusted for sex and age. The effect of smoking on the risk of the sequence variants rs4662344-T in ARHGAP15, rs7609897-T in COLQ and rs67153654-A in FAM155A was also calculated.

Effect of inflammatory biomarkers.

The Data on inflammatory biomarkers were obtained from three of the largest laboratories in Iceland (measurements performed between the years 1993 and 2015): the laboratory of the Landspitali University Hospital, the Icelandic Medical Center (Laeknasetrid) Laboratory in Mjodd (RAM) and Akureyri Hospital (FSA). The following data were used: C reactive protein (CRP) levels (N=202,790), white blood cell count (N=273,110), neutrophil count (N=252,010), and erythrocyte sedimentation rate (N=176,966). All blood measurements were standardized using quantile-quantile standardization and then corrected for year of birth (or age), sex and age at measurement[8].

Sample preparation and DNA whole-genome sequencing methods.

Our dataset contains samples obtained using three different library preparation methods from Illumina. In addition sequencing was performed using three different types of Illumina sequencing instruments

a) Standard TruSeq DNA library preparation method. Illumina GAIIx and/or HiSeq 2000 sequencers.

b) TruSeq DNA PCR-free library preparation method. Illumina HiSeq 2500 sequencers.

c) TruSeq Nano DNA library preparation method. Illumina HiSeq X sequencers.

A more detailed description of each sample preparation method is provided below.

Sample preparation and sequencing using the standard TruSeq DNA library preparation method.

Approximately 1 µg of genomic DNA, isolated from frozen blood samples, was fragmented to a mean target size of approximately 300-400 bp using a Covaris E210 instrument. The resulting fragmented DNA was end repaired using T4 and Klenow polymerases and T4 polynucleotide kinase with 10 mM dNTP followed by addition of an 'A' base at the ends using Klenow exo fragment (3’ to 5’-exo minus) and dATP (1 mM). Sequencing adaptors containing 'T' overhangs were ligated to the DNA products followed by agarose (2%) gel electrophoresis. Fragments of about 450-500 bp were isolated from the gels (QIAGEN Gel Extraction Kit), and the adaptor-modified DNA fragments were PCR enriched for ten cycles using Phusion DNA polymerase (Finnzymes Oy) and a PCR primer cocktail needed for paired-end sequencing. Enriched libraries were purified using AMPure XP beads. The quality and concentration of the

libraries were assessed with the Agilent 2100 Bioanalyzer using the DNA 1000 LabChip. Libraries were stored at −20 °C. Sequencing-by-synthesis (SBS) was performed on either Illumina GAIIx or HiSeq 2000 instruments, respectively. Paired-end libraries were sequenced using 2x76, 2x101 or 2x120 cycles of incorporation and imaging with Illumina SBS kits, TruSeq™ v5 for the GAIIx. For the HiSeq 2000, 2x101 cycles with SBS kits v2.5 or v3 were employed. Each library was initially run on a single lane on a GAIIx for validation, assessing optimal cluster densities, insert size, duplication rates and comparison to chip genotyping data. Following validation, the desired sequencing depth (10X to 30X) was then obtained using either sequencing platform. Targeted raw cluster densities ranged from 500–800 K/mm2 , depending on the version of both the sequencing chemistry and the data imaging/analysis software packages (SCS.2.8/RTA1.8 or SCS2.9/RTA1.9 for the GAIIx and HCS1.3.8. or HCS1.4.8 for HiSeq 2000). Real-time analysis involved conversion of image data to base-calling in real-time.

Sample preparation and sequencing using the TruSeq DNA PCR-free method.

Paired-end libraries for sequencing were prepared according to the manufacturer's instructions (Illumina, TruSeq DNA PCR-free ™). In short, approximately 1 µg of genomic DNA, isolated from frozen blood samples, was fragmented to Nature Genetics: doi:10.1038/ng.3816 9 a mean target size of 350 bp using a Covaris E210 ultrasonicator followed by clean-up using AmPure XP purification beads. Blunt-end DNA from the resulting fragments was generated using a mix of 3’>5’ exonuclease and 5’>3’ polymerase activities, respectively, followed by 5’-phosphorylation using T4 polynucleotide kinase. Size-selection of the blunt-end fragments was done using a two-step purification strategy with different ratios of the AmPure XP purification beads (0.6X and 1X). Finally, 3’-adenylation and ligation of barcoded adapters was performed, followed by clean-up with magnetic beads. The quality and concentration of the libraries were assessed with the Agilent 2100 Bioanalyzer using the DNA 1000 LabChip (Agilent). Barcoded libraries were stored at −20 °C. All steps in the workflow were monitored using an in-house laboratory information management system with barcode tracking of all samples and reagents. All samples were first pooled (12-24 plex) and sequenced on Illumina´s MiSeq instruments (2x25 cycles) to assess quality and effective concentration of sequencing libraries. Subsequent deep sequencing was done on HiSeq 2500 instruments, were each sample was sequenced on 3 lanes, generating >100 Gb of raw data and at least 30X coverage. Sequencing was done using TruSeq v3 reagents, paired-end 2x100 cycles. System operation and base calling in real-time was done using HCS 2.2.38 and RTA 1.18.61.

Sample preparation and sequencing using the TruSeq Nano DNA method.

The sample preparation workflow was essentially the same as described above for the TruSeq DNA PCR-free method, except the input amount was 100 ng of genomic DNA (instead of 1 μg) and following clean-up of adapter ligated DNA, the samples were enriched by 8-cycles of PCR using a PCR primer cocktail, followed by Ampure XP bead clean-up. The quality and concentration of the libraries were assessed with the Perkin Elmer LabChip GX instrument using the HT DNA HiSens reagent kit. Sequencing was done using the HiSeq X HD reagent kit. Each sample was loaded onto the HiSeq X instrument at a concentration of 300 pM and sequenced to high depth (>30X). System operation and base calling in real-time was done using HCSX 3.1.26 and RTA2 2.3.9.

References.

1. Sveinbjornsson G*, et al.* Weighting sequence variants based on their annotation increases power of whole-genome association studies. *Nature genetics* **48**, 314-317 (2016).

2. Liu JZ*, et al.* Association analyses identify 38 susceptibility loci for inflammatory bowel disease and highlight shared genetic risk across populations. *Nature genetics* **47**, 979-986 (2015).

3. Ward LD, Kellis M. HaploReg v4: systematic mining of putative causal variants, cell types, regulators and target genes for human complex traits and disease. *Nucleic acids research* **44**, D877-881 (2016).

4. Grainger S, Savory JG, Lohnes D. Cdx2 regulates patterning of the intestinal epithelium. *Dev Biol* **339**, 155-165 (2010).

5. Kakizaki F, Aoki K, Miyoshi H, Carrasco N, Aoki M, Taketo MM. CDX transcription factors positively regulate expression of solute carrier family 5, member 8 in the colonic epithelium. *Gastroenterology* **138**, 627-635 (2010).

6. Thorgeirsson TE*, et al.* Sequence variants at CHRNB3-CHRNA6 and CYP2A6 affect smoking behavior. *Nature genetics* **42**, 448-453 (2010).

7. Thorgeirsson TE*, et al.* A rare missense mutation in CHRNA4 associates with smoking behavior and its consequences. *Mol Psychiatry* **21**, 594-600 (2016).

8. Gudbjartsson DF*, et al.* Large-scale whole-genome sequencing of the Icelandic population. *Nature genetics* **47**, 435-444 (2015).
